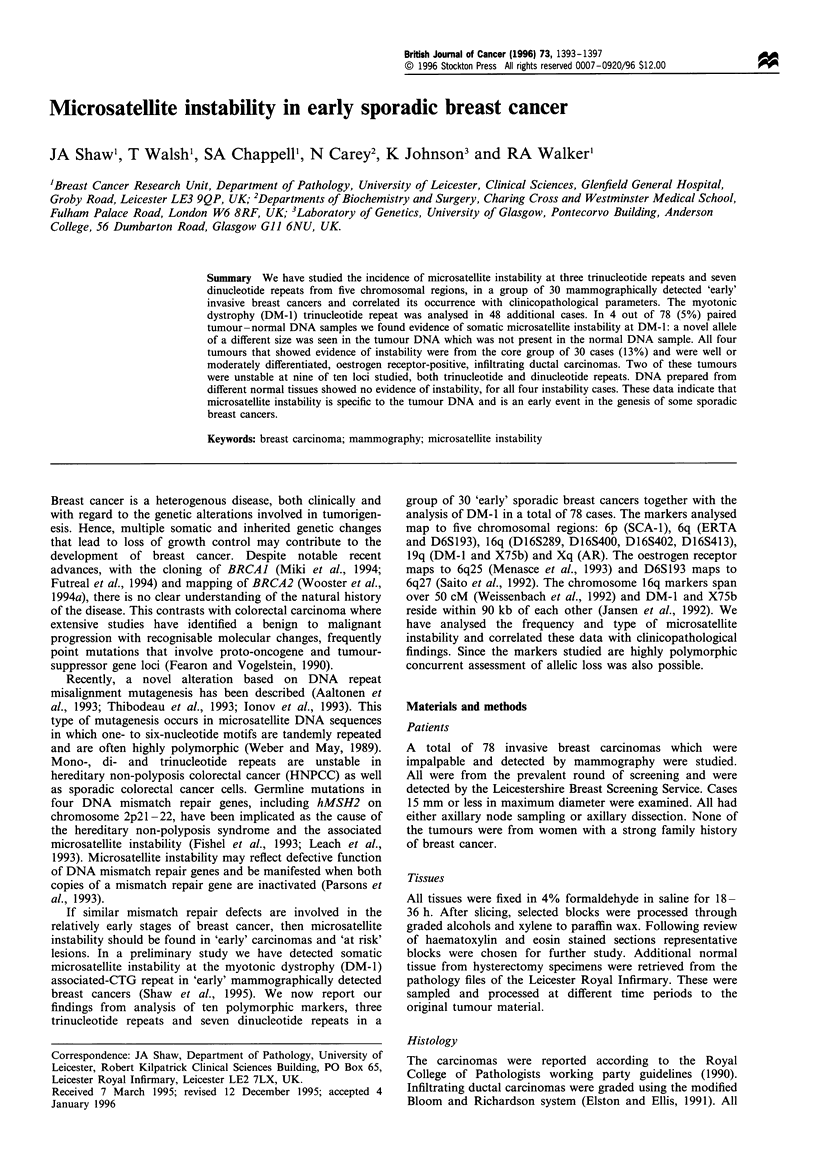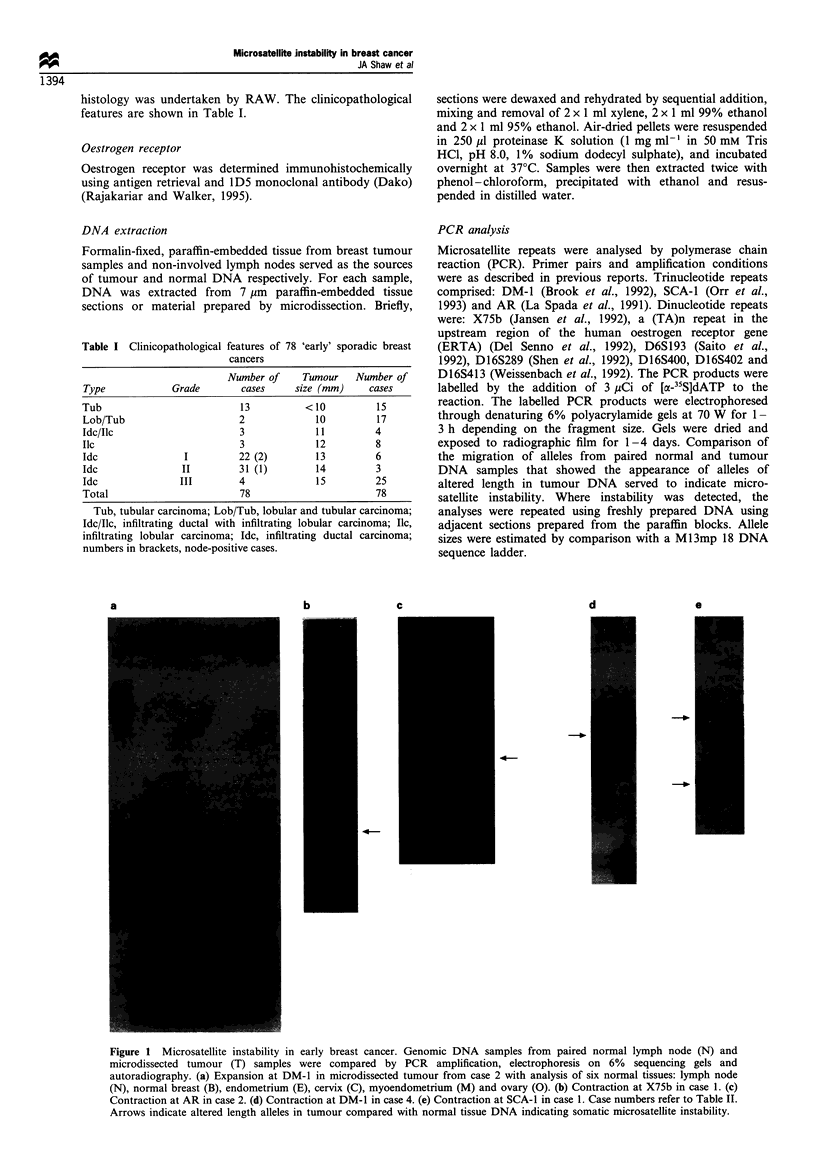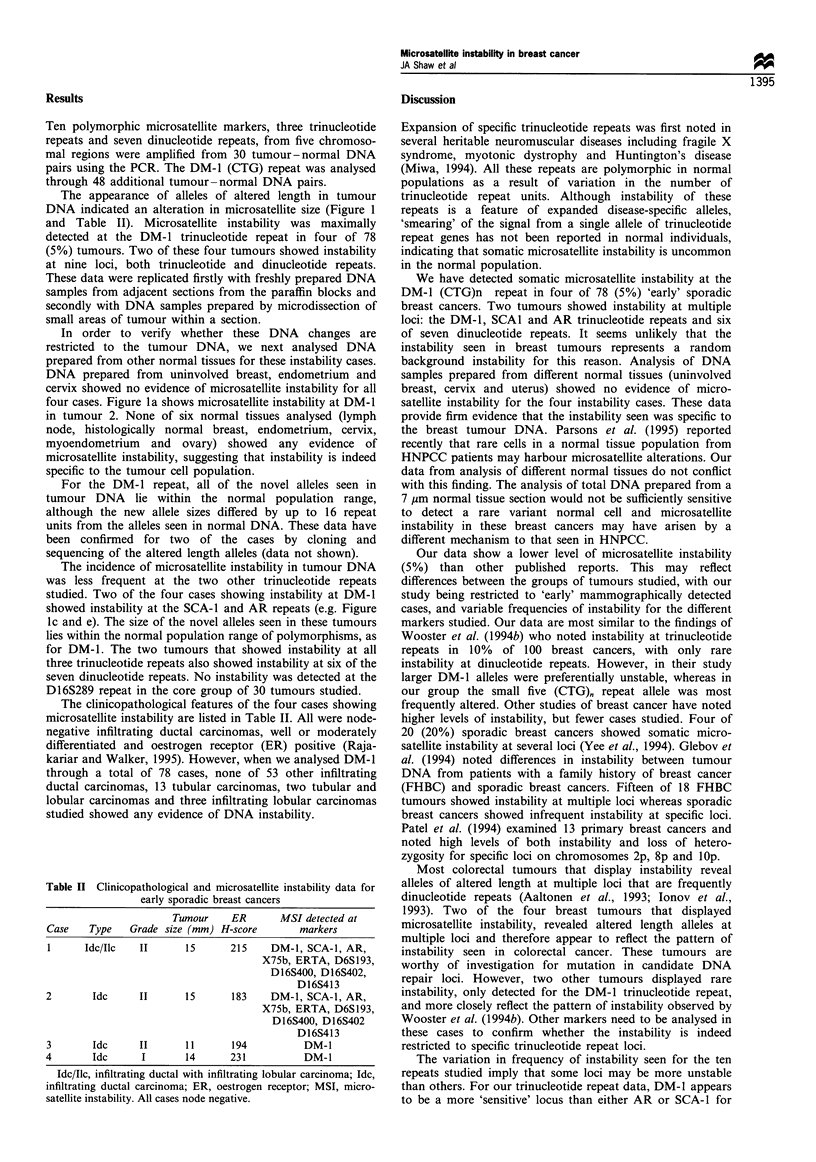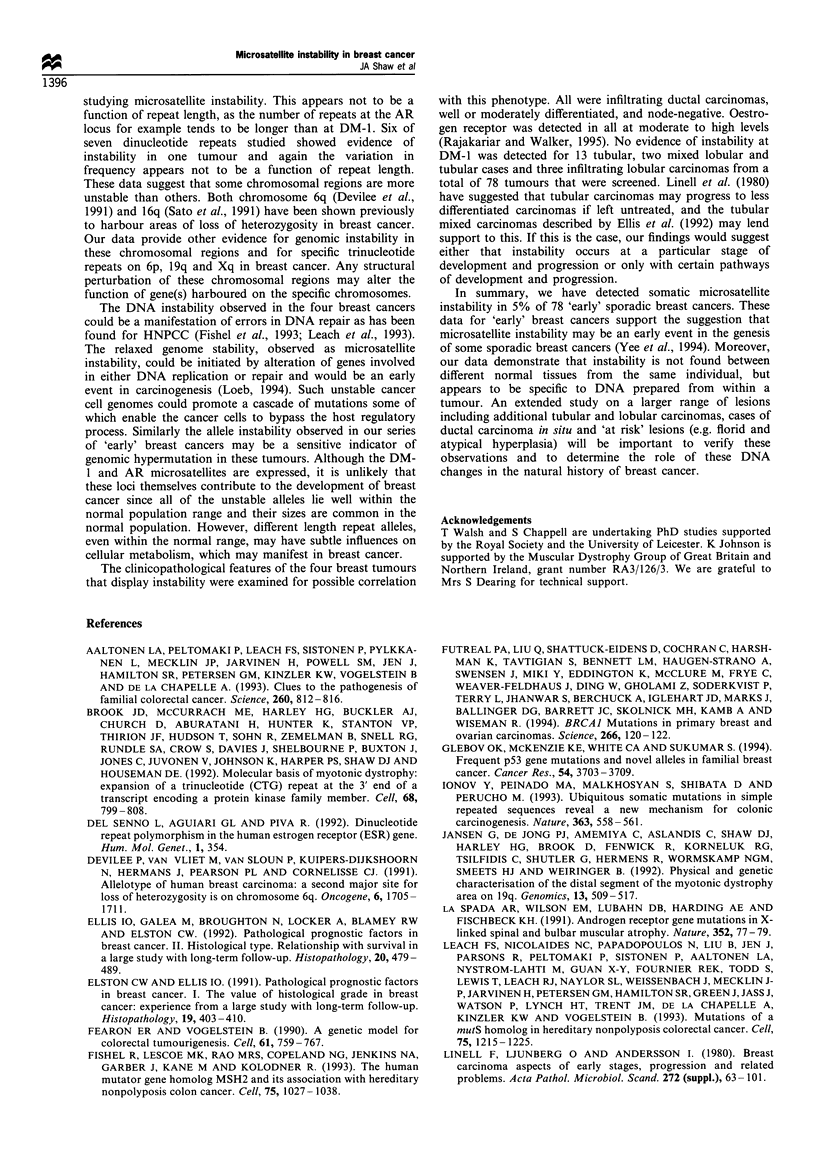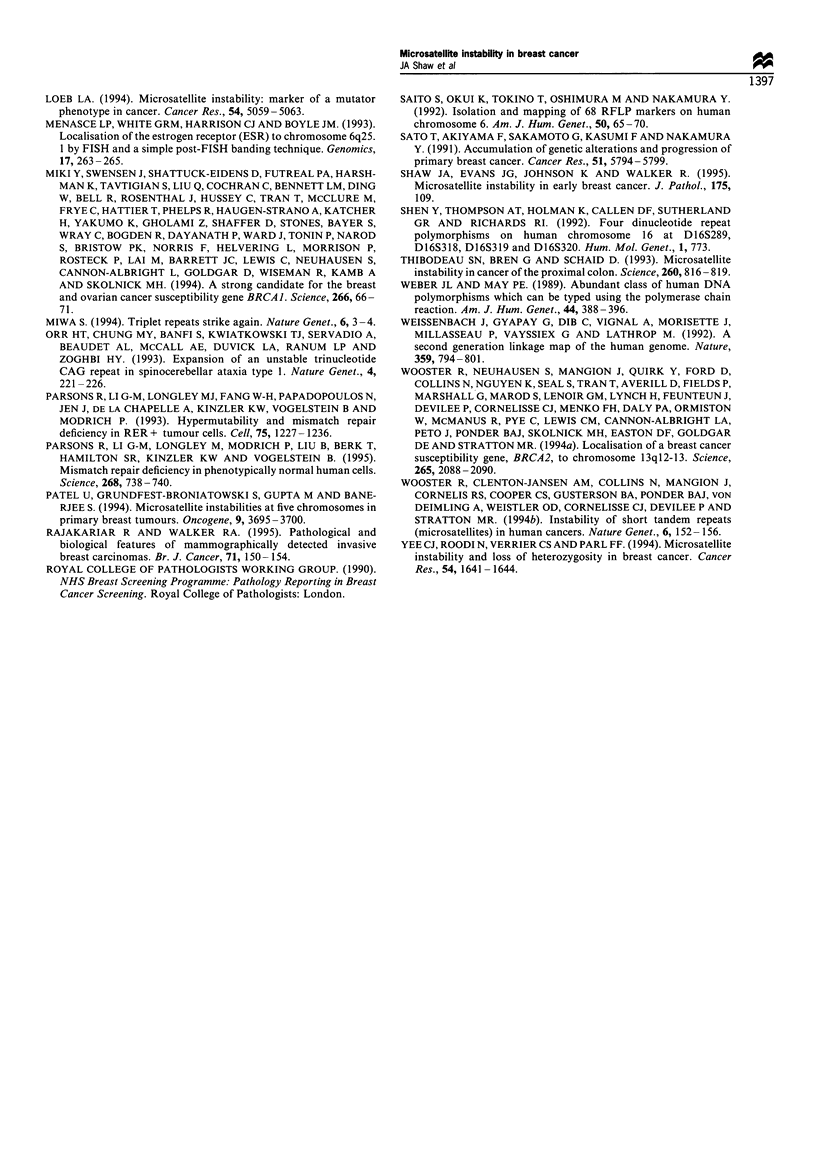# Microsatellite instability in early sporadic breast cancer.

**DOI:** 10.1038/bjc.1996.264

**Published:** 1996-06

**Authors:** J. A. Shaw, T. Walsh, S. A. Chappell, N. Carey, K. Johnson, R. A. Walker

**Affiliations:** Department of Pathology, University of Leicester, Glenfield General Hospital, UK.

## Abstract

**Images:**


British Journal of Cancer (1996) 73, 1393-1397
© 1996 Stockton Press All rights reserved 0007-0920/96 $12.00

### Microsatellite instability in early sporadic breast cancer

JA Shaw[1], T Walsh[1], SA Chappell[1], N Carey[2], K Johnson[3] and RA Walker[1]

[1]*Breast Cancer Research Unit, Department of Pathology, University of Leicester, Clinical Sciences, Glenfield General Hospital, Groby Road, Leicester LE3 9QP, UK;* [2]*Departments of Biochemistry and Surgery, Charing Cross and Westminster Medical School, Fulham Palace Road, London W6 8RF, UK;* [3]*Laboratory of Genetics, University of Glasgow, Pontecorvo Building, Anderson College, 56 Dumbarton Road, Glasgow G11 6NU, UK.*

**Summary** We have studied the incidence of microsatellite instability at three trinucleotide repeats and seven dinucleotide repeats from five chromosomal regions, in a group of 30 mammographically detected 'early' invasive breast cancers and correlated its occurrence with clinicopathological parameters. The myotonic dystrophy (DM-1) trinucleotide repeat was analysed in 48 additional cases. In 4 out of 78 (5%) paired tumour-normal DNA samples we found evidence of somatic microsatellite instability at DM-1: a novel allele of a different size was seen in the tumour DNA which was not present in the normal DNA sample. All four tumours that showed evidence of instability were from the core group of 30 cases (13%) and were well or moderately differentiated, oestrogen receptor-positive, infiltrating ductal carcinomas. Two of these tumours were unstable at nine of ten loci studied, both trinucleotide and dinucleotide repeats. DNA prepared from different normal tissues showed no evidence of instability, for all four instability cases. These data indicate that microsatellite instability is specific to the tumour DNA and is an early event in the genesis of some sporadic breast cancers.

**Keywords:** breast carcinoma; mammography; microsatellite instability

Breast cancer is a heterogenous disease, both clinically and with regard to the genetic alterations involved in tumorigenesis. Hence, multiple somatic and inherited genetic changes that lead to loss of growth control may contribute to the development of breast cancer. Despite notable recent advances, with the cloning of *BRCA1* (Miki *et al.*, 1994; Futreal *et al.*, 1994) and mapping of *BRCA2* (Wooster *et al.*, 1994*a*), there is no clear understanding of the natural history of the disease. This contrasts with colorectal carcinoma where extensive studies have identified a benign to malignant progression with recognisable molecular changes, frequently point mutations that involve proto-oncogene and tumour-suppressor gene loci (Fearon and Vogelstein, 1990).

Recently, a novel alteration based on DNA repeat misalignment mutagenesis has been described (Aaltonen *et al.*, 1993; Thibodeau *et al.*, 1993; Ionov *et al.*, 1993). This type of mutagenesis occurs in microsatellite DNA sequences in which one- to six-nucleotide motifs are tandemly repeated and are often highly polymorphic (Weber and May, 1989). Mono-, di- and trinucleotide repeats are unstable in hereditary non-polyposis colorectal cancer (HNPCC) as well as sporadic colorectal cancer cells. Germline mutations in four DNA mismatch repair genes, including *hMSH2* on chromosome 2p21-22, have been implicated as the cause of the hereditary non-polyposis syndrome and the associated microsatellite instability (Fishel *et al.*, 1993; Leach *et al.*, 1993). Microsatellite instability may reflect defective function of DNA mismatch repair genes and be manifested when both copies of a mismatch repair gene are inactivated (Parsons *et al.*, 1993).

If similar mismatch repair defects are involved in the relatively early stages of breast cancer, then microsatellite instability should be found in 'early' carcinomas and 'at risk' lesions. In a preliminary study we have detected somatic microsatellite instability at the myotonic dystrophy (DM-1) associated-CTG repeat in 'early' mammographically detected breast cancers (Shaw *et al.*, 1995). We now report our findings from analysis of ten polymorphic markers, three trinucleotide repeats and seven dinucleotide repeats in a group of 30 'early' sporadic breast cancers together with the analysis of DM-1 in a total of 78 cases. The markers analysed map to five chromosomal regions: 6p (SCA-1), 6q (ERTA and D6S193), 16q (D16S289, D16S400, D16S402, D16S413), 19q (DM-1 and X75b) and Xq (AR). The oestrogen receptor maps to 6q25 (Menasce *et al.*, 1993) and D6S193 maps to 6q27 (Saito *et al.*, 1992). The chromosome 16q markers span over 50 cM (Weissenbach *et al.*, 1992) and DM-1 and X75b reside within 90 kb of each other (Jansen *et al.*, 1992). We have analysed the frequency and type of microsatellite instability and correlated these data with clinicopathological findings. Since the markers studied are highly polymorphic concurrent assessment of allelic loss was also possible.

### Materials and methods

### *Patients*

A total of 78 invasive breast carcinomas which were impalpable and detected by mammography were studied. All were from the prevalent round of screening and were detected by the Leicestershire Breast Screening Service. Cases 15 mm or less in maximum diameter were examined. All had either axillary node sampling or axillary dissection. None of the tumours were from women with a strong family history of breast cancer.

### *Tissues*

All tissues were fixed in 4% formaldehyde in saline for 18-36 h. After slicing, selected blocks were processed through graded alcohols and xylene to paraffin wax. Following review of haematoxylin and eosin stained sections representative blocks were chosen for further study. Additional normal tissue from hysterectomy specimens were retrieved from the pathology files of the Leicester Royal Infirmary. These were sampled and processed at different time periods to the original tumour material.

### *Histology*

The carcinomas were reported according to the Royal College of Pathologists working party guidelines (1990). Infiltrating ductal carcinomas were graded using the modified Bloom and Richardson system (Elston and Ellis, 1991). All

Correspondence: JA Shaw, Department of Pathology, University of Leicester, Robert Kilpatrick Clinical Sciences Building, PO Box 65, Leicester Royal Infirmary, Leicester LE2 7LX, UK.
Received 7 March 1995; revised 12 December 1995; accepted 4 January 1996

histology was undertaken by RAW. The clinicopathological features are shown in Table I.

### Oestrogen receptor

Oestrogen receptor was determined immunohistochemically using antigen retrieval and 1D5 monoclonal antibody (Dako) (Rajakariar and Walker, 1995).

### DNA extraction

Formalin-fixed, paraffin-embedded tissue from breast tumour samples and non-involved lymph nodes served as the sources of tumour and normal DNA respectively. For each sample, DNA was extracted from 7 $\mu$m paraffin-embedded tissue sections or material prepared by microdissection. Briefly, sections were dewaxed and rehydrated by sequential addition, mixing and removal of $2 \times 1$ ml xylene, $2 \times 1$ ml 99% ethanol and $2 \times 1$ ml 95% ethanol. Air-dried pellets were resuspended in 250 $\mu$l proteinase K solution (1 mg $ml^{-1}$ in 50 mM Tris HCl, pH 8.0, 1% sodium dodecyl sulphate), and incubated overnight at 37°C. Samples were then extracted twice with phenol–chloroform, precipitated with ethanol and resuspended in distilled water.

**Table I** Clinicopathological features of 78 'early' sporadic breast cancers

| *Type* | *Grade* | *Number of cases* | *Tumour size (mm)* | *Number of cases* |
|---|---|---|---|---|
| Tub | | 13 | <10 | 15 |
| Lob/Tub | | 2 | 10 | 17 |
| Idc/Ilc | | 3 | 11 | 4 |
| Ilc | | 3 | 12 | 8 |
| Idc | I | 22 (2) | 13 | 6 |
| Idc | II | 31 (1) | 14 | 3 |
| Idc | III | 4 | 15 | 25 |
| Total | | 78 | | 78 |

Tub, tubular carcinoma; Lob/Tub, lobular and tubular carcinoma; Idc/Ilc, infiltrating ductal with infiltrating lobular carcinoma; Ilc, infiltrating lobular carcinoma; Idc, infiltrating ductal carcinoma; numbers in brackets, node-positive cases.

### PCR analysis

Microsatellite repeats were analysed by polymerase chain reaction (PCR). Primer pairs and amplification conditions were as described in previous reports. Trinucleotide repeats comprised: DM-1 (Brook *et al.*, 1992), SCA-1 (Orr *et al.*, 1993) and AR (La Spada *et al.*, 1991). Dinucleotide repeats were: X75b (Jansen *et al.*, 1992), a (TA)n repeat in the upstream region of the human oestrogen receptor gene (ERTA) (Del Senno *et al.*, 1992), D6S193 (Saito *et al.*, 1992), D16S289 (Shen *et al.*, 1992), D16S400, D16S402 and D16S413 (Weissenbach *et al.*, 1992). The PCR products were labelled by the addition of 3 $\mu$Ci of [$\alpha$-$^{35}$S]dATP to the reaction. The labelled PCR products were electrophoresed through denaturing 6% polyacrylamide gels at 70 W for 1–3 h depending on the fragment size. Gels were dried and exposed to radiographic film for 1–4 days. Comparison of the migration of alleles from paired normal and tumour DNA samples that showed the appearance of alleles of altered length in tumour DNA served to indicate microsatellite instability. Where instability was detected, the analyses were repeated using freshly prepared DNA using adjacent sections prepared from the paraffin blocks. Allele sizes were estimated by comparison with a M13mp 18 DNA sequence ladder.

**Figure 1** Microsatellite instability in early breast cancer. Genomic DNA samples from paired normal lymph node (N) and microdissected tumour (T) samples were compared by PCR amplification, electrophoresis on 6% sequencing gels and autoradiography. (**a**) Expansion at DM-1 in microdissected tumour from case 2 with analysis of six normal tissues: lymph node (N), normal breast (B), endometrium (E), cervix (C), myoendometrium (M) and ovary (O). (**b**) Contraction at X75b in case 1. (**c**) Contraction at AR in case 2. (**d**) Contraction at DM-1 in case 4. (**e**) Contraction at SCA-1 in case 1. Case numbers refer to Table II. Arrows indicate altered length alleles in tumour compared with normal tissue DNA indicating somatic microsatellite instability.

### Results

Ten polymorphic microsatellite markers, three trinucleotide repeats and seven dinucleotide repeats, from five chromosomal regions were amplified from 30 tumour–normal DNA pairs using the PCR. The DM-1 (CTG) repeat was analysed through 48 additional tumour–normal DNA pairs.

The appearance of alleles of altered length in tumour DNA indicated an alteration in microsatellite size (Figure 1 and Table II). Microsatellite instability was maximally detected at the DM-1 trinucleotide repeat in four of 78 (5%) tumours. Two of these four tumours showed instability at nine loci, both trinucleotide and dinucleotide repeats. These data were replicated firstly with freshly prepared DNA samples from adjacent sections from the paraffin blocks and secondly with DNA samples prepared by microdissection of small areas of tumour within a section.

In order to verify whether these DNA changes are restricted to the tumour DNA, we next analysed DNA prepared from other normal tissues for these instability cases. DNA prepared from uninvolved breast, endometrium and cervix showed no evidence of microsatellite instability for all four cases. Figure 1a shows microsatellite instability at DM-1 in tumour 2. None of six normal tissues analysed (lymph node, histologically normal breast, endometrium, cervix, myoendometrium and ovary) showed any evidence of microsatellite instability, suggesting that instability is indeed specific to the tumour cell population.

For the DM-1 repeat, all of the novel alleles seen in tumour DNA lie within the normal population range, although the new allele sizes differed by up to 16 repeat units from the alleles seen in normal DNA. These data have been confirmed for two of the cases by cloning and sequencing of the altered length alleles (data not shown).

The incidence of microsatellite instability in tumour DNA was less frequent at the two other trinucleotide repeats studied. Two of the four cases showing instability at DM-1 showed instability at the SCA-1 and AR repeats (e.g. Figure 1c and e). The size of the novel alleles seen in these tumours lies within the normal population range of polymorphisms, as for DM-1. The two tumours that showed instability at all three trinucleotide repeats also showed instability at six of the seven dinucleotide repeats. No instability was detected at the D16S289 repeat in the core group of 30 tumours studied.

The clinicopathological features of the four cases showing microsatellite instability are listed in Table II. All were node-negative infiltrating ductal carcinomas, well or moderately differentiated and oestrogen receptor (ER) positive (Rajakariar and Walker, 1995). However, when we analysed DM-1 through a total of 78 cases, none of 53 other infiltrating ductal carcinomas, 13 tubular carcinomas, two tubular and lobular carcinomas and three infiltrating lobular carcinomas studied showed any evidence of DNA instability.

**Table II** Clinicopathological and microsatellite instability data for early sporadic breast cancers

| *Case* | *Type* | *Grade* | *Tumour size (mm)* | *ER H-score* | *MSI detected at markers* |
|---|---|---|---|---|---|
| 1 | Idc/Ilc | II | 15 | 215 | DM-1, SCA-1, AR, X75b, ERTA, D6S193, D16S400, D16S402, D16S413 |
| 2 | Idc | II | 15 | 183 | DM-1, SCA-1, AR, X75b, ERTA, D6S193, D16S400, D16S402 D16S413 |
| 3 | Idc | II | 11 | 194 | DM-1 |
| 4 | Idc | I | 14 | 231 | DM-1 |

Idc/Ilc, infiltrating ductal with infiltrating lobular carcinoma; Idc, infiltrating ductal carcinoma; ER, oestrogen receptor; MSI, microsatellite instability. All cases node negative.

### Discussion

Expansion of specific trinucleotide repeats was first noted in several heritable neuromuscular diseases including fragile X syndrome, myotonic dystrophy and Huntington's disease (Miwa, 1994). All these repeats are polymorphic in normal populations as a result of variation in the number of trinucleotide repeat units. Although instability of these repeats is a feature of expanded disease-specific alleles, 'smearing' of the signal from a single allele of trinucleotide repeat genes has not been reported in normal individuals, indicating that somatic microsatellite instability is uncommon in the normal population.

We have detected somatic microsatellite instability at the DM-1 (CTG)n repeat in four of 78 (5%) 'early' sporadic breast cancers. Two tumours showed instability at multiple loci: the DM-1, SCA1 and AR trinucleotide repeats and six of seven dinucleotide repeats. It seems unlikely that the instability seen in breast tumours represents a random background instability for this reason. Analysis of DNA samples prepared from different normal tissues (uninvolved breast, cervix and uterus) showed no evidence of microsatellite instability for the four instability cases. These data provide firm evidence that the instability seen was specific to the breast tumour DNA. Parsons *et al.* (1995) reported recently that rare cells in a normal tissue population from HNPCC patients may harbour microsatellite alterations. Our data from analysis of different normal tissues do not conflict with this finding. The analysis of total DNA prepared from a 7 $\mu$m normal tissue section would not be sufficiently sensitive to detect a rare variant normal cell and microsatellite instability in these breast cancers may have arisen by a different mechanism to that seen in HNPCC.

Our data show a lower level of microsatellite instability (5%) than other published reports. This may reflect differences between the groups of tumours studied, with our study being restricted to 'early' mammographically detected cases, and variable frequencies of instability for the different markers studied. Our data are most similar to the findings of Wooster *et al.* (1994*b*) who noted instability at trinucleotide repeats in 10% of 100 breast cancers, with only rare instability at dinucleotide repeats. However, in their study larger DM-1 alleles were preferentially unstable, whereas in our group the small five $(CTG)_n$ repeat allele was most frequently altered. Other studies of breast cancer have noted higher levels of instability, but fewer cases studied. Four of 20 (20%) sporadic breast cancers showed somatic microsatellite instability at several loci (Yee *et al.*, 1994). Glebov *et al.* (1994) noted differences in instability between tumour DNA from patients with a family history of breast cancer (FHBC) and sporadic breast cancers. Fifteen of 18 FHBC tumours showed instability at multiple loci whereas sporadic breast cancers showed infrequent instability at specific loci. Patel *et al.* (1994) examined 13 primary breast cancers and noted high levels of both instability and loss of heterozygosity for specific loci on chromosomes 2p, 8p and 10p.

Most colorectal tumours that display instability reveal alleles of altered length at multiple loci that are frequently dinucleotide repeats (Aaltonen *et al.*, 1993; Ionov *et al.*, 1993). Two of the four breast tumours that displayed microsatellite instability, revealed altered length alleles at multiple loci and therefore appear to reflect the pattern of instability seen in colorectal cancer. These tumours are worthy of investigation for mutation in candidate DNA repair loci. However, two other tumours displayed rare instability, only detected for the DM-1 trinucleotide repeat, and more closely reflect the pattern of instability observed by Wooster *et al.* (1994*b*). Other markers need to be analysed in these cases to confirm whether the instability is indeed restricted to specific trinucleotide repeat loci.

The variation in frequency of instability seen for the ten repeats studied imply that some loci may be more unstable than others. For our trinucleotide repeat data, DM-1 appears to be a more 'sensitive' locus than either AR or SCA-1 for studying microsatellite instability. This appears not to be a function of repeat length, as the number of repeats at the AR locus for example tends to be longer than at DM-1. Six of seven dinucleotide repeats studied showed evidence of instability in one tumour and again the variation in frequency appears not to be a function of repeat length. These data suggest that some chromosomal regions are more unstable than others. Both chromosome 6q (Devilee et al., 1991) and 16q (Sato et al., 1991) have been shown previously to harbour areas of loss of heterozygosity in breast cancer. Our data provide other evidence for genomic instability in these chromosomal regions and for specific trinucleotide repeats on 6p, 19q and Xq in breast cancer. Any structural perturbation of these chromosomal regions may alter the function of gene(s) harboured on the specific chromosomes.

The DNA instability observed in the four breast cancers could be a manifestation of errors in DNA repair as has been found for HNPCC (Fishel et al., 1993; Leach et al., 1993). The relaxed genome stability, observed as microsatellite instability, could be initiated by alteration of genes involved in either DNA replication or repair and would be an early event in carcinogenesis (Loeb, 1994). Such unstable cancer cell genomes could promote a cascade of mutations some of which enable the cancer cells to bypass the host regulatory process. Similarly the allele instability observed in our series of 'early' breast cancers may be a sensitive indicator of genomic hypermutation in these tumours. Although the DM-1 and AR microsatellites are expressed, it is unlikely that these loci themselves contribute to the development of breast cancer since all of the unstable alleles lie well within the normal population range and their sizes are common in the normal population. However, different length repeat alleles, even within the normal range, may have subtle influences on cellular metabolism, which may manifest in breast cancer.

The clinicopathological features of the four breast tumours that display instability were examined for possible correlation with this phenotype. All were infiltrating ductal carcinomas, well or moderately differentiated, and node-negative. Oestrogen receptor was detected in all at moderate to high levels (Rajakariar and Walker, 1995). No evidence of instability at DM-1 was detected for 13 tubular, two mixed lobular and tubular cases and three infiltrating lobular carcinomas from a total of 78 tumours that were screened. Linell et al. (1980) have suggested that tubular carcinomas may progress to less differentiated carcinomas if left untreated, and the tubular mixed carcinomas described by Ellis et al. (1992) may lend support to this. If this is the case, our findings would suggest either that instability occurs at a particular stage of development and progression or only with certain pathways of development and progression.

In summary, we have detected somatic microsatellite instability in 5% of 78 'early' sporadic breast cancers. These data for 'early' breast cancers support the suggestion that microsatellite instability may be an early event in the genesis of some sporadic breast cancers (Yee et al., 1994). Moreover, our data demonstrate that instability is not found between different normal tissues from the same individual, but appears to be specific to DNA prepared from within a tumour. An extended study on a larger range of lesions including additional tubular and lobular carcinomas, cases of ductal carcinoma *in situ* and 'at risk' lesions (e.g. florid and atypical hyperplasia) will be important to verify these observations and to determine the role of these DNA changes in the natural history of breast cancer.

**Acknowledgements**
T Walsh and S Chappell are undertaking PhD studies supported by the Royal Society and the University of Leicester. K Johnson is supported by the Muscular Dystrophy Group of Great Britain and Northern Ireland, grant number RA3/126/3. We are grateful to Mrs S Dearing for technical support.

### References

AALTONEN LA, PELTOMAKI P, LEACH FS, SISTONEN P, PYLKKANEN L, MECKLIN JP, JARVINEN H, POWELL SM, JEN J, HAMILTON SR, PETERSEN GM, KINZLER KW, VOGELSTEIN B AND DE LA CHAPELLE A. (1993). Clues to the pathogenesis of familial colorectal cancer. *Science*, **260**, 812–816.

BROOK JD, MCCURRACH ME, HARLEY HG, BUCKLER AJ, CHURCH D, ABURATANI H, HUNTER K, STANTON VP, THIRION JF, HUDSON T, SOHN R, ZEMELMAN B, SNELL RG, RUNDLE SA, CROW S, DAVIES J, SHELBOURNE P, BUXTON J, JONES C, JUVONEN V, JOHNSON K, HARPER PS, SHAW DJ AND HOUSEMAN DE. (1992). Molecular basis of myotonic dystrophy: expansion of a trinucleotide (CTG) repeat at the 3′ end of a transcript encoding a protein kinase family member. *Cell*, **68**, 799–808.

DEL SENNO L, AGUIARI GL AND PIVA R. (1992). Dinucleotide repeat polymorphism in the human estrogen receptor (ESR) gene. *Hum. Mol. Genet.*, **1**, 354.

DEVILEE P, VAN VLIET M, VAN SLOUN P, KUIPERS-DIJKSHOORN N, HERMANS J, PEARSON PL AND CORNELISSE CJ. (1991). Allelotype of human breast carcinoma: a second major site for loss of heterozygosity is on chromosome 6q. *Oncogene*, **6**, 1705–1711.

ELLIS IO, GALEA M, BROUGHTON N, LOCKER A, BLAMEY RW AND ELSTON CW. (1992). Pathological prognostic factors in breast cancer. II. Histological type. Relationship with survival in a large study with long-term follow-up. *Histopathology*, **20**, 479–489.

ELSTON CW AND ELLIS IO. (1991). Pathological prognostic factors in breast cancer. I. The value of histological grade in breast cancer: experience from a large study with long-term follow-up. *Histopathology*, **19**, 403–410.

FEARON ER AND VOGELSTEIN B. (1990). A genetic model for colorectal tumourigenesis. *Cell*, **61**, 759–767.

FISHEL R, LESCOE MK, RAO MRS, COPELAND NG, JENKINS NA, GARBER J, KANE M AND KOLODNER R. (1993). The human mutator gene homolog MSH2 and its association with hereditary nonpolyposis colon cancer. *Cell*, **75**, 1027–1038.

FUTREAL PA, LIU Q, SHATTUCK-EIDENS D, COCHRAN C, HARSHMAN K, TAVTIGIAN S, BENNETT LM, HAUGEN-STRANO A, SWENSEN J, MIKI Y, EDDINGTON K, MCCLURE M, FRYE C, WEAVER-FELDHAUS J, DING W, GHOLAMI Z, SODERKVIST P, TERRY L, JHANWAR S, BERCHUCK A, IGLEHART JD, MARKS J, BALLINGER DG, BARRETT JC, SKOLNICK MH, KAMB A AND WISEMAN R. (1994). *BRCA1* Mutations in primary breast and ovarian carcinomas. *Science*, **266**, 120–122.

GLEBOV OK, MCKENZIE KE, WHITE CA AND SUKUMAR S. (1994). Frequent p53 gene mutations and novel alleles in familial breast cancer. *Cancer Res.*, **54**, 3703–3709.

IONOV Y, PEINADO MA, MALKHOSYAN S, SHIBATA D AND PERUCHO M. (1993). Ubiquitous somatic mutations in simple repeated sequences reveal a new mechanism for colonic carcinogenesis. *Nature*, **363**, 558–561.

JANSEN G, DE JONG PJ, AMEMIYA C, ASLANDIS C, SHAW DJ, HARLEY HG, BROOK D, FENWICK R, KORNELUK RG, TSILFIDIS C, SHUTLER G, HERMENS R, WORMSKAMP NGM, SMEETS HJ AND WEIRINGER B. (1992). Physical and genetic characterisation of the distal segment of the myotonic dystrophy area on 19q. *Genomics*, **13**, 509–517.

LA SPADA AR, WILSON EM, LUBAHN DB, HARDING AE AND FISCHBECK KH. (1991). Androgen receptor gene mutations in X-linked spinal and bulbar muscular atrophy. *Nature*, **352**, 77–79.

LEACH FS, NICOLAIDES NC, PAPADOPOULOS N, LIU B, JEN J, PARSONS R, PELTOMAKI P, SISTONEN P, AALTONEN LA, NYSTROM-LAHTI M, GUAN X-Y, FOURNIER REK, TODD S, LEWIS T, LEACH RJ, NAYLOR SL, WEISSENBACH J, MECKLIN J-P, JARVINEN H, PETERSEN GM, HAMILTON SR, GREEN J, JASS J, WATSON P, LYNCH HT, TRENT JM, DE LA CHAPELLE A, KINZLER KW AND VOGELSTEIN B. (1993). Mutations of a *mut*S homolog in hereditary nonpolyposis colorectal cancer. *Cell*, **75**, 1215–1225.

LINELL F, LJUNBERG O AND ANDERSSON I. (1980). Breast carcinoma aspects of early stages, progression and related problems. *Acta Pathol. Microbiol. Scand.* **272 (suppl.)**, 63–101.

LOEB LA. (1994). Microsatellite instability: marker of a mutator phenotype in cancer. *Cancer Res.*, **54**, 5059–5063.

MENASCE LP, WHITE GRM, HARRISON CJ AND BOYLE JM. (1993). Localisation of the estrogen receptor (ESR) to chromosome 6q25. 1 by FISH and a simple post-FISH banding technique. *Genomics*, **17**, 263–265.

MIKI Y, SWENSEN J, SHATTUCK-EIDENS D, FUTREAL PA, HARSHMAN K, TAVTIGIAN S, LIU Q, COCHRAN C, BENNETT LM, DING W, BELL R, ROSENTHAL J, HUSSEY C, TRAN T, MCCLURE M, FRYE C, HATTIER T, PHELPS R, HAUGEN-STRANO A, KATCHER H, YAKUMO K, GHOLAMI Z, SHAFFER D, STONES, BAYER S, WRAY C, BOGDEN R, DAYANATH P, WARD J, TONIN P, NAROD S, BRISTOW PK, NORRIS F, HELVERING L, MORRISON P, ROSTECK P, LAI M, BARRETT JC, LEWIS C, NEUHAUSEN S, CANNON-ALBRIGHT L, GOLDGAR D, WISEMAN R, KAMB A AND SKOLNICK MH. (1994). A strong candidate for the breast and ovarian cancer susceptibility gene *BRCA1*. *Science*, **266**, 66–71.

MIWA S. (1994). Triplet repeats strike again. *Nature Genet.*, **6**, 3–4.

ORR HT, CHUNG MY, BANFI S, KWIATKOWSKI TJ, SERVADIO A, BEAUDET AL, MCCALL AE, DUVICK LA, RANUM LP AND ZOGHBI HY. (1993). Expansion of an unstable trinucleotide CAG repeat in spinocerebellar ataxia type 1. *Nature Genet.*, **4**, 221–226.

PARSONS R, LI G-M, LONGLEY MJ, FANG W-H, PAPADOPOULOS N, JEN J, DE LA CHAPELLE A, KINZLER KW, VOGELSTEIN B AND MODRICH P. (1993). Hypermutability and mismatch repair deficiency in RER+ tumour cells. *Cell*, **75**, 1227–1236.

PARSONS R, LI G-M, LONGLEY M, MODRICH P, LIU B, BERK T, HAMILTON SR, KINZLER KW AND VOGELSTEIN B. (1995). Mismatch repair deficiency in phenotypically normal human cells. *Science*, **268**, 738–740.

PATEL U, GRUNDFEST-BRONIATOWSKI S, GUPTA M AND BANERJEE S. (1994). Microsatellite instabilities at five chromosomes in primary breast tumours. *Oncogene*, **9**, 3695–3700.

RAJAKARIAR R AND WALKER RA. (1995). Pathological and biological features of mammographically detected invasive breast carcinomas. *Br. J. Cancer*, **71**, 150–154.

ROYAL COLLEGE OF PATHOLOGISTS WORKING GROUP. (1990). *NHS Breast Screening Programme: Pathology Reporting in Breast Cancer Screening*. Royal College of Pathologists: London.

SAITO S, OKUI K, TOKINO T, OSHIMURA M AND NAKAMURA Y. (1992). Isolation and mapping of 68 RFLP markers on human chromosome 6. *Am. J. Hum. Genet.*, **50**, 65–70.

SATO T, AKIYAMA F, SAKAMOTO G, KASUMI F AND NAKAMURA Y. (1991). Accumulation of genetic alterations and progression of primary breast cancer. *Cancer Res.*, **51**, 5794–5799.

SHAW JA, EVANS JG, JOHNSON K AND WALKER R. (1995). Microsatellite instability in early breast cancer. *J. Pathol.*, **175**, 109.

SHEN Y, THOMPSON AT, HOLMAN K, CALLEN DF, SUTHERLAND GR AND RICHARDS RI. (1992). Four dinucleotide repeat polymorphisms on human chromosome 16 at D16S289, D16S318, D16S319 and D16S320. *Hum. Mol. Genet.*, **1**, 773.

THIBODEAU SN, BREN G AND SCHAID D. (1993). Microsatellite instability in cancer of the proximal colon. *Science*, **260**, 816–819.

WEBER JL AND MAY PE. (1989). Abundant class of human DNA polymorphisms which can be typed using the polymerase chain reaction. *Am. J. Hum. Genet.*, **44**, 388–396.

WEISSENBACH J, GYAPAY G, DIB C, VIGNAL A, MORISETTE J, MILLASSEAU P, VAYSSIEX G AND LATHROP M. (1992). A second generation linkage map of the human genome. *Nature*, **359**, 794–801.

WOOSTER R, NEUHAUSEN S, MANGION J, QUIRK Y, FORD D, COLLINS N, NGUYEN K, SEAL S, TRAN T, AVERILL D, FIELDS P, MARSHALL G, MAROD S, LENOIR GM, LYNCH H, FEUNTEUN J, DEVILEE P, CORNELISSE CJ, MENKO FH, DALY PA, ORMISTON W, MCMANUS R, PYE C, LEWIS CM, CANNON-ALBRIGHT LA, PETO J, PONDER BAJ, SKOLNICK MH, EASTON DF, GOLDGAR DE AND STRATTON MR. (1994*a*). Localisation of a breast cancer susceptibility gene, *BRCA2*, to chromosome 13q12-13. *Science*, **265**, 2088–2090.

WOOSTER R, CLENTON-JANSEN AM, COLLINS N, MANGION J, CORNELIS RS, COOPER CS, GUSTERSON BA, PONDER BAJ, VON DEIMLING A, WEISTLER OD, CORNELISSE CJ, DEVILEE P AND STRATTON MR. (1994*b*). Instability of short tandem repeats (microsatellites) in human cancers. *Nature Genet.*, **6**, 152–156.

YEE CJ, ROODI N, VERRIER CS AND PARL FF. (1994). Microsatellite instability and loss of heterozygosity in breast cancer. *Cancer Res.*, **54**, 1641–1644.